# Tropomyosin Isoforms Segregate into Distinct Clusters on Single Actin Filaments

**DOI:** 10.3390/biom14101240

**Published:** 2024-09-30

**Authors:** Peyman Obeidy, Thomas Sobey, Philip R. Nicovich, Adelle C. F. Coster, Elvis Pandzic

**Affiliations:** 1 Discipline of Medical Imaging Science, School of Health Sciences, Faculty of Medicine and Health, The University of Sydney, Sydney, NSW 2006, Australia; 2 Single Molecule Science, School of Medical Sciences, University of New South Wales, Sydney, NSW 2052, Australia; t.sobey@unsw.edu.au; 3 Allen Institute for Brain Science, Seattle, WA 98109, USA; p.nicovich@cajalneuro.com; 4 School of Mathematics & Statistics, Faculty of Science, University of New South Wales, Sydney, NSW 2052, Australia; a.coster@unsw.edu.au; 5 Katharina Gaus Light Microscopy Facility, Mark Wainwright Analytical Centre, University of New South Wales, Sydney, NSW 2052, Australia

**Keywords:** tropomyosin, single actin filament, TIRF microscopy, assembly, automated image analysis algorithm

## Abstract

Tropomyosins (Tpms) are rod-shaped proteins that interact head-to-tail to form a continuous polymer along both sides of most cellular actin filaments. Head-to-tail interaction between adjacent Tpm molecules and the formation of an overlap complex between them leads to the assembly of actin filaments with one type of Tpm isoform in time and space. Variations in the affinity of tropomyosin isoforms for different actin structures are proposed as a potential sorting mechanism. However, the detailed mechanisms of the spatio-temporal sorting of Tpms remain elusive. In this study, we investigated the early intermediates during actin–tropomyosin filament assembly, using a skeletal/cardiac Tpm isoform (Tpm1.1) and a cytoskeletal isoform (Tpm1.6) that differ only in the last 27 amino acids. We investigated how the muscle isoform Tpm1.1 and the cytoskeletal isoform Tpm1.6 nucleate domains on the actin filament, and tested whether (1) recruitment is affected by the actin isoform (muscle vs. cytoskeletal) and (2) whether there is specificity in recruiting the same isoform to a domain at these early stages. To address these questions, actin filaments were exposed to low concentrations of fluorescent tropomyosins in solution. The filaments were immobilized onto glass coverslips and the pattern of decoration was visualized by TIRF microscopy. We show that at the early assembly stage, tropomyosins formed multiple distinct fluorescent domains (here termed “cluster”) on the actin filaments. An automated image analysis algorithm was developed and validated to identify clusters and estimate the number of tropomyosins in each cluster. The analysis showed that tropomyosin isoform sorting onto an actin filament is unlikely to be driven by a preference for nucleating on the corresponding muscle or cytoskeletal actin isoforms, but rather is facilitated by a higher probability of incorporating the same tropomyosin isoforms into an early assembly intermediate. We showed that the 27 amino acids at the end of each tropomyosin seem to provide enough molecular information for the attachment of the same tropomyosin isoforms adjacent to each other on an actin filament. This results in the formation of homogeneous clusters composed of the same isoform rather than clusters with mixed isoforms.

## 1. Introduction

Tropomyosins (Tpms) form co-polymers with actin and are composed of a family of over 40 isoforms. Cytoskeletal tropomyosins (Tpms) primarily form homodimers, which subsequently assemble into homopolymers with actin, contributing to the compositional diversity of actin filaments [1,2]. Their interaction with actin leads to the specialization of the actin filament function in time and space [3]. Due to alternative splicing, most tropomyosin molecules have differentially encoded N- and C-termini. The overlap region plays an important role in determining the orientation and dynamics of tropomyosin dimers on filamentous actin [4]. Multiple studies have established that the N- and C-terminal overlapping regions are not functionally equivalent [5]. Differences in these sequences affect the binding of tropomyosin to actin, as well as that of other actin-binding proteins [6,7]. The current Tpm/F-actin binding model supports tropomyosin molecules interacting head-to-tail to form a homogeneous continuous coiled-coil structure floating in the positively charged major groove of an actin filament [2,8]. This assembly model also postulates that cooperativity in tropomyosin binding is driven by an 8–11 amino acid overlap between the amino and carboxyl termini of adjacent tropomyosin dimers [9]. Although it has been shown that the overlap regions could lead to distinct functions for Tpms [5], the detailed mechanism of this interaction remains elusive. To elucidate the intrinsic mechanism of tropomyosin sorting to the actin filament, we employed a single-molecule imaging approach based on total internal reflection fluorescence (TIRF) microscopy and microfluidics. We investigate whether the amino acid differences between the labeled Tpms are sufficient to guide and discriminate between these isoforms during the early stages of tropomyosin assembly. We selected the tropomyosin dimers, Tpm1.1 and Tpm1.6, which are identical in their amino acid sequences apart from their C-termini (last 27 amino acids), which are encoded by different exons [2].

In a typical TIRF setup, light is reflected at an interface between two media with different refractive indices. It generates an evanescent field that effectively produces a ~150 nm-thick exponentially decaying excitation profile [10]. Although restricting excitation to a very thin section near the coverslip allows single-molecule detection, a lack of automated tools for quantification and resolving the number of molecules within sub-diffraction limited clusters remains challenging. Here, we combine single-molecule detection with the use of microfluidic devices to facilitate and automate image processing to investigate the early assembly of tropomyosins bound to actin filaments. We deconvolved and counted the number of tropomyosins in bright diffraction-limited clusters based on photobleaching [11] and intensity distribution-based deconvolving approaches [12,13].

In this paper we report that the segregation of Tpm isoforms may be intrinsic to different proteins. We have visualized and quantified snapshots of the assembly process by reconstituting actin filament in the presence of labeled cytoskeletal (Tpm1.6) and skeletal (Tpm1.1) Tpm isoforms, which bind to different sites on actin filaments. We used the closely related recombinant tropomyosin isoforms from the TPM1 gene, skeletal–muscle Tpm1.1 and cytoskeletal Tpm1.6 isoforms. Our findings show that at low concentrations, Tpms (1.1 and 1.6) bind multiple locations along naked *α*- and *β*- actin filaments to form small clusters. No intrinsic preferences were observed for the sorting of the correct Tpm to its biologically relevant actin filament; for example, Tpm1.6 binds with higher affinity than Tpm1.1 to both actin isoforms.

## 2. Materials and Methods

### 2.1. Expression, Purification, and Labeling of Tpms

The expression of recombinant human Tpm1.6 (Tm2) and rat Tpm1.1 (αTm) with N-terminal Ala-Ser extension in *E. coli* BL21 (DE3) cells using pET3a+ expression vectors was carried out as described previously [14]. The protein was purified using ammonium sulfate fractionation, dialysis, chromatography, and gel filtration chromatography, and then lyophilized for storage at −20 °C as described previously [15]. The lyophilized protein was resuspended in 1–2 mL T buffer (150 mM NaCl, 10 mM Tris-HCl pH 7.5, 2 mM MgCl_2_) supplemented with 5 mM DTT and then dialyzed against T buffer containing 0.5 mM DTT (2 × 2 h). The Cys-190 residues of the Tpm1.6 and Tpm1.1 dimers are prone to form a disulfide. Thus, the protein was reduced prior to labeling by incubation in the presence of 20 mM DTT at 37 °C for 2 h. To remove the DTT, the buffer was then exchanged with T buffer using desalting columns (Zeba Microspin, 7k MWCO, Thermo Fisher Scientific, Cleveland, OH, USA). Tpms were then reacted at Cys-190 with a five-fold molar excess of Alexa Fluor 488-C5-maleimide (Ex/Em: 495/519 nm) or Alexa Fluor 568-C5-maleimide (Ex/Em: 578/603 nm) (Life Technologies, 5791 Van Allen Way, Carlsbad, CA 92008, USA) in the dark at 37 °C for 2 h. The reaction was quenched using β-mercaptoethanol added at a two-fold molar excess over the dye. Finally, the unconjugated dye was removed using Zeba columns (up to five times). The purity of the labeled mixture was examined using SDS-PAGE with fluorescence detection (UV excitation) and Coomassie Blue staining. The labeled protein concentration was measured using the Direct Detect spectrometer (Merck Millipore, Germany). The degree of labeling was calculated as the ratio of the dye concentration and the protein concentration, whereby the dye concentration was determined from the absorption of the sample at the wavelength corresponding to the maximum absorption for the dye and the extinction coefficient.

### 2.2. Co-Sedimentation of Tropomyosin-Actin

The affinities of unlabeled and labeled tropomyosins for actin filaments were measured using a standard co-sedimentation assay [15]. Prior to co-sedimentation, tropomyosin isoforms were incubated with 1 mM DTT and heated in a water bath at 56 °C for 5 min. Increasing concentrations of tropomyosins (0.2–3.5 µM) were mixed and incubated with 3 µM rabbit skeletal–muscle F-actin (Cytoskeleton Inc., CO, USA) in T buffer at a final reaction volume of 50 µL for 1 h at room temperature. Following the incubation, the samples were spun in a bench-top Beckman ultracentrifuge at 670,000× *g* at 20 °C for 30 min (TLA-120.1 rotor, Beckman-Coulter, 5350 Lakeview Parkway S Drive, Indianapolis, IN 46268, USA) to pellet F-actin and associated Tpm. Supernatant and pellet fractions were resolved on a 12% polyacrylamide gel; proteins were visualized by Coomassie Blue staining and the protein bands were quantified by densitometry (Epson Perfection V750 Pro scanner, Long Beach, CA, USA and ImageJ (Version 1.48), Wayne Rasband, National Institutes of Health). The ratio of the Tpm/actin density was plotted as a function of the concentration of free Tpm in the supernatant determined by densitometry. The Hill equation (Equation (1)) was used to fit the binding curves:

v = n [Tpm]^h^/((Kd(app))^h^ + [Tpm]^h^) (1)

where v is the Tpm/actin density ratio, [Tpm] is the concentration of free Tpm, n is the maximum Tpm/actin density ratio, h is the Hill coefficient and Kd(app) is the apparent equilibrium dissociation constant for Tpm binding to F-actin [15].

### 2.3. Single-Molecule Photobleaching

Freshly cleaned coverslips were clamped into a magnetic chamber (Chamlide CMB, Live Cell Instruments, Namyangju, Republic of Korea), and labeled tropomyosins (Tpm1.6 AF488, Tpm1.6 AF568, Tpm1.1 AF488, and Tpm1.1 AF568) were sparsely adsorbed to the coverslips from solution (32 pM) in TIR50 buffer (50 mM NaCl, 10 mM Tris-HCl pH 7.8, 2 mM MgCl_2_, 1 mM ATP, 0.2 mM CaCl_2_ and 50 mM DTT) supplemented with additional NaCl to a final concentration of 150 mM to prevent oligomerization. After incubation (5 min), the surface was rinsed and the solution in the chamber was replaced with 200 µL of TIR50 buffer.

Tropomyosin dimers appeared as a diffraction-limited spot in the time-lapse images collected using Total Internal Reflection Florescence (TIRF) microscopy. Images were collected at an imaging rate of 5 frames per second until most of the spots were photobleached. Spots with two bleaching steps (corresponding to the two fluorophores on a tropomyosin dimer) were selected and fitted with a 2D Gaussian function to determine the amplitude and half-width (sigma, σ) of the diffraction-limited signals. Mean values for amplitude and σ were determined by fitting the corresponding histograms of the data (~100 photobleaching events) with a Gaussian distribution.

### 2.4. Preparation of Microfluidic Flow Cells

Coverslips (Marienfeld Superior, VWR, No. 1.5H, 24 mm × 60 mm, Lauda-Königshofen, Germany) were sonicated in acetone for 5 min followed by rinsing with Milli-Q water. Filtered N_2_ gas was blown over the coverslip before passing the coverslip briefly through the flame of a Bunsen burner. The dried coverslip was treated in a plasma cleaner for 3 min at a pressure of ~650 Torr. Microfluidic devices with channel dimensions of 1 × 0.8 × 0.04 mm (*L* × *W* × *H*) were prepared from polydimethylsiloxane (PDMS) using replica molding and cured in an oven at 70 °C for 2 h. The PDMS device was punctured at the channel ends with a biopsy punch (1 mm diameter) for connecting the device to tubing, and the device was then cleaned with isopropanol. The PDMS device was placed onto a freshly plasma-cleaned coverslip and adhered together by placing the assembled device under a weight (600 g) for 3 min. Finally, polyethylene tubing (PE20 Intramedic, Clay Adams, the internal diameter of 0.38 mm) was inserted into the holes at the inlet and outlet of the channels in the assembled device.

### 2.5. The Capture of Actin Filaments on Modified Coverslip Surfaces

The microfluidic channels were filled with a solution of a copolymer of poly-l-lysine (PLL) and poly (ethylene glycol) (PEG) (Susos AG, PLL (20)-g[3.4]-PEG (2)/PEG (3.4)-biotin (20%)) in PBS (5 µL, 1 mg mL^−1^) and the copolymer was allowed to adsorb to the glass surface for 20 min at room temperature. The unbound PLL-PEG was washed out of the channel with 20 µL of PBS. The coated surface was then treated with blocking buffer (20 mM Tris pH 7.5, 2 mM EDTA, 50 mM NaCl, 0.03% NaN_3_, 0.025% Tween 20, 0.2 mg/mL BSA) at room temperature in a humid chamber for 10 min. The outlet tubing of the PDMS device was connected to a syringe pump operated in withdraw mode, and each channel was subsequently rinsed with 20 µL of PBS followed by 20 µL of 5% BSA, incubated for 10 min and subsequently washed with 20 µL of D buffer (10 mM Tris-HCl pH 7.8, 2 mM MgCl_2_, 1 mM ATP, 0.2 mM CaCl_2_ and 50 mM DTT). This surface was used for the non-specific capture of actin filaments decorated with labeled tropomyosin. Labeled tropomyosins (0.015 µM of each isoform) were mixed prior to adding 1 µM monomeric actin (G-actin, Hypermol, Bielefeld, German) in D buffer. Actin polymerization was triggered by adding 50 mM NaCl and incubating the mixture for 5–10 min. Subsequently, the reaction mixture was gently mixed with a solution of polyethylene glycol (PEG 300) to a final concentration of 10% *v*/*v*, just before injecting 3–6 µL of the mixture into the microfluidic chambers. The channel was then rinsed with TIR50 buffer at a flow rate of 5 µL/min. Decorated filaments were allowed to bind to the surface and unbound filaments were removed by washing the microfluidic channel with TIR50 buffer at a flow rate of 5 µL/min with 20 µL.

### 2.6. TIRF Imaging of Labeled Tpm1.1 and Tpm1.6 Binding to Actin Filament

Single-molecule experiments were conducted using an inverted TIRF microscope (TILL Photonics, Scitech, Victoria, Australia) with Andor iXon3 897 Ultra back-illuminated EM-CCD cameras (Hamamatsu, Spectra Services, Ontario, NY, USA) and 100X/1.46 Alpha-Plan apochromatic Oil 0.17 mm (UV) Vis-IR objective (Carl Zeiss Microscopy, NY, USA). Images were acquired using the TILL Photonics microscopy software packages Live Acquisition (LA) and Offline Analysis (OA). Dual-color time-lapse TIRF imaging was conducted using 488 nm (Dynamic Laser, Salt Lake City, UT, USA) and 568 nm (Coherent, Santa Clara, CA, USA) lasers at 20% (~1 mW) laser power, a multiplication gain setting of 300, 100 ms laser exposure time and an imaging frequency of 5 Hz.

### 2.7. Calibrating Tropomyosin Numbers on an Actin Filament

Clusters of tropomyosin dimers bound to actin filaments appeared as punctate signals in the TIRF images. Tropomyosin clusters were fitted with the 2D Gaussian function and then iteratively deconvolved from the overlapping clusters, until the background level was reached. The number of fluorophores in each cluster was then calculated by dividing the total integral intensity of the diffraction-limited spot of a cluster by that of a single fluorophore. For simplicity, we will refer to the total integral intensity of a diffraction-limited spot as a volume. An automated MATLAB algorithm (see Supplementary Materials) was used to fit clusters on each filament and calculate the total number of fluorophores per filament. The algorithm was validated on datasets with filaments with a sparse pattern of labeled tropomyosins and using simulated cluster data. The algorithm was designed for processing a large number of filaments from independent experiments and distributions of cluster sizes (number of fluorophores per cluster) and the distances between clusters to be determined. The following steps were taken to correct these misidentifications: (a) peaks within a distance of 2*σ* pixels of each other, equivalent to the standard deviation, *σ*, of the point-spread function (PSF), (b) small-intensity peaks (with *σ* < 1) remaining after this process were assigned to noise, as it is physically impossible to have PSF with *σ* < 1 pixel, and excluded from further analysis, (c) peaks with intensity value equivalent to a *σ*/2 or smaller and more than three pixels were excluded, (d) filaments with only one type of tropomyosin (usually small filament) or only one of each tropomyosin were also excluded.

## 3. Results

### 3.1. Labeled Tropomyosins Bind Cooperatively to Actin Filaments

**Figure 1:**
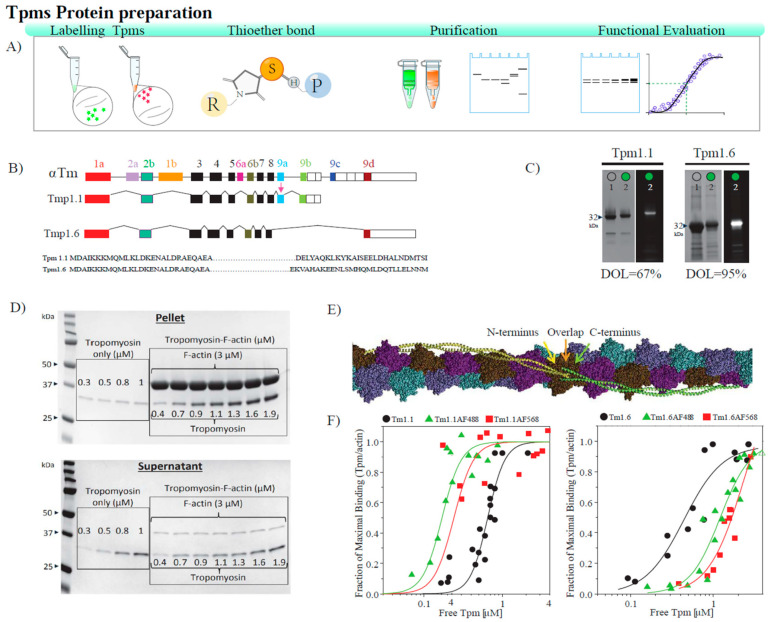
Labeled tropomyosins bind cooperatively to actin filaments. (**A**) Schematic illustration of Tpms preparation, labeling and functional evaluation. (**B**) Diagram of Tpm1.1 (αTm) and Tpm1.6 (Tm2) isoforms. (**C**) Cysteine residues at position 190 on the recombinant tropomyosin dimers were labeled with Alexa Fluor 488 (ε = 71,000) or 568 (ε = 91,300) C5 maleimide. Image adapted from [17]. (**C**) Representative SDS-PAGE with Coomassie Blue staining (left) and fluorescence detection (right). Lane 1, unlabeled protein; lane 2, a protein labeled with Alexa Fluor 488 C5 maleimide dye. Western blot original images can be found in Supplementary Materials. (**D**) SDS-PAGE gels of the (bottom panel) supernatant and (top panel) pellet fractions from sedimentation of Tpms with filamentous α-actin stained with Coomassie Blue. (**E**) Tpms interact head-to-tail on the actin filament. (**F**) Binding curves were obtained from co-sedimentation of unlabeled and labeled (right panel) Tpm1.1 and (left panel) Tpm1.6 to skeletal–muscle F-actin. The muscle F-actin data come from three separate experiments, except for Tpm1.6, which used data from two experiments. Data points were combined from three independent experiments except for Tpm1.6 n = 2. The (**B**,**E**) images were adapted from Gunning et al., 2008, Orzechowski et al. (2014), respectively [2,18].

Both Tpm1.1 and Tpm1.6 isoforms, derived from the TPM1 gene, were labeled on their only cysteine at position 190 using maleimide derivatives of Alexa Fluor 488 (AF488) and 568 (AF568) (Figure 1A,B). These Tpm isoforms contain a few aromatic amino acids and have a relatively low extinction coefficient, 5960 and 8940 cm^−1^M^−1^, respectively. Thus, we calculated the degree of labeling ratio by dividing the concentration of the fluorophore (measured using UV light absorption) in the labeled protein by the concentration of the protein measured using (infrared (IR) absorption). The monomer labeling ratio was determined to be approximately 67 and 95% for Tpm1.1 and Tpm1.6, respectively (Figure 1C). Assuming a random incorporation of the dye, this labeling ratio corresponds to a mixture containing Tpm dimers labeled with two, one or no fluorophores, whereby singly labeled dimers constitute the main fraction. Subsequently, we evaluated the impact of the labeling process on the binding of labeled Tpm1.1 and Tpm1.6 to skeletal–muscle F-actin using the standard co-sedimentation assay (Figure 1D,E). Our findings show that the Alexa Flour 488 (AF488) and 568 (AF568) maleimide labeling of Tpm1.1 and Tpm1.6 did not affect the formation of Tpm-actin filaments, nor was the cooperative nature of the binding affected (Table 1). These results agree with previously reported studies of the retention of the binding ability of Tpm1.1 containing modified 190 cysteine residues [16].

### 3.2. Single-Molecule Calibration Based on Photobleaching

**Figure 2:**
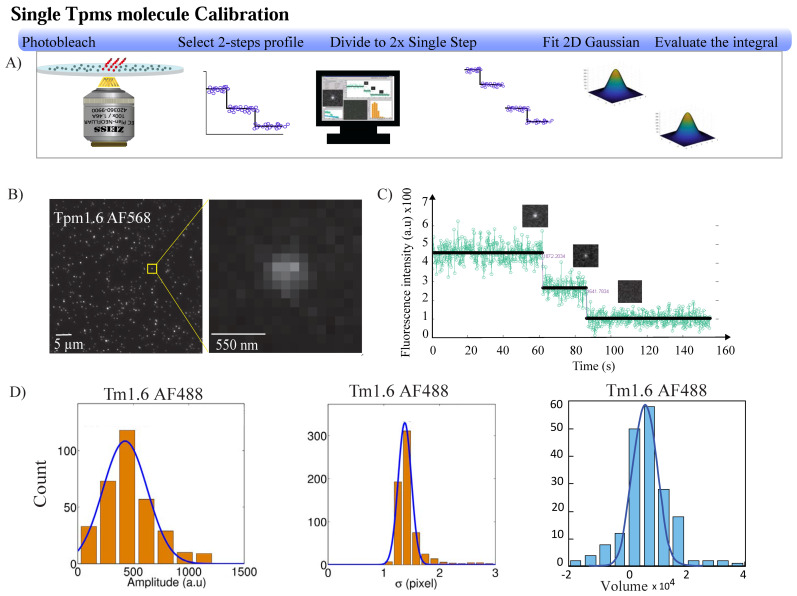
Single-molecule intensity calibration based on photobleaching. (**A**) Schematic illustration of Tpms calibration as a single molecule. (**B**) Representative TIRF image of labeled tropomyosin adhered to the coverslip. Each labeled molecule appears as a diffraction-limited spot. (**C**) Stepwise photobleaching of a diffraction-limited spot as a function of time. (**D**) Distributions of the fluorescence amplitudes (left panel). Standard deviation (σ) (center panel) and total integral (volume) of detected spot (right panel) for the fitted Gaussian profiles of the single molecule (see Table 2).

To calibrate the properties of single-labeled Tpms, we collected the data for the diffraction-limited spots with one- and two-step photobleaching profiles. The two-step photobleaching profile was broken into two single-step profiles before pooling the values. Photobleaching traces were generated by fitting the fluorescent spot in each frame with a 2D symmetric Gaussian function and calculating its volume using Equation (2);

(2) $$V=2\pi A\sigma^{2}$$

where A represents the amplitude (height of the Gaussian function above the background intensity) and σ is the standard deviation. The intensity of a single fluorophore was then determined as the step height between intensity levels in the photobleaching profiles. The mean intensity and standard deviation of a single fluorophore were determined by fitting the distribution of step heights with a Gaussian function (Figure 2D). The data are summarized in Table 2. We further determined the number of fluorophores for each spot present in the first frame of the fluorescence movie used initially for the photobleaching analysis. The volume of the spot was divided by the volume of a single fluorophore. The resulting distribution confirmed that 81% of the Tpm dimers were labeled with a single fluorophore. These data were also consistent with the aforementioned labeling ratio determined by spectroscopy.

### 3.3. Observation and Quantification of Actin–Tropomyosin Filament Assembly at the Single-Molecule Level

**Figure 3:**
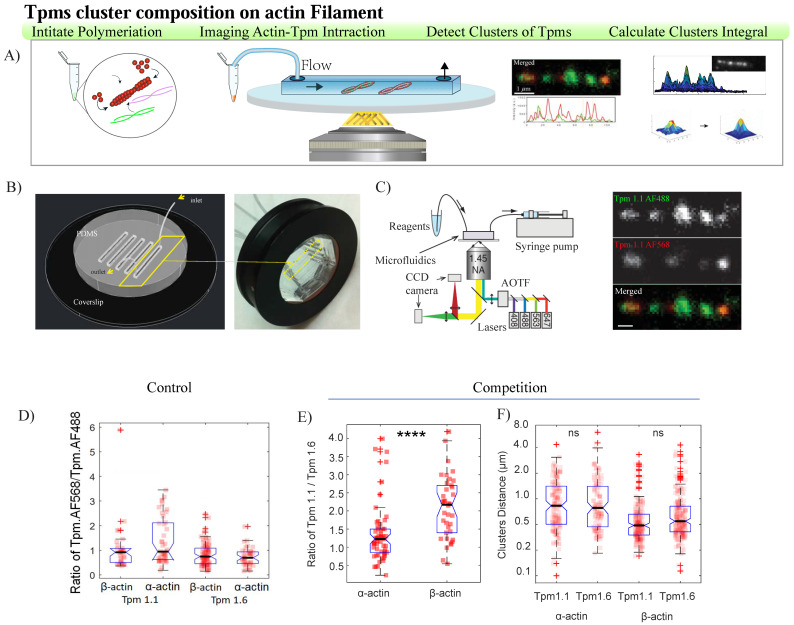
Observation and quantification of tropomyosin molecules bound to actin filaments assembly at the single-molecule level using a TIRF microscope configuration and flow-cell design. Schematic illustration of the capturing of decorated filaments to the surface. (**A**) Actin filaments are decorated with the fluorescent Tpm molecules in solution and then injected into the flow channel of the microfluidic imaging device for capture on the chemically modified coverslip. (**B**) PDMS device assembly. (**C**) Fluorophores captured on the filament are excited with laser light, which can be modulated with an acoustic–optical tunable filter (AOTF). The emission light is collected via a high-numerical-aperture objective and detected with a sensitive electron-multiplying charge-coupled device (CCD) camera. The emission path includes a beam splitter for simultaneous dual-color imaging (left panel). The representative images of actin filaments are decorated with labeled tropomyosins (right panel). (**D**) The distribution of the same tropomyosin isoforms’ ratio is labeled with two different fluorophores on actin filaments (control experiments). Actin (1 μM) was co-assembled with tropomyosin (0.015 μM of each label) prior to the capture of the decorated filament. The distributions of the ratio of the different tropomyosin isoforms labeled with two different fluorophores are shown on actin filaments (competition experiments, (**E**). Data for Tpm1.1AF488 and Tpm1.1.AF568 pooled into Tpm1.1, and similarly for Tmp1.6. The distribution between consecutive clusters on actin filaments in a competition experiment (**F**). Data are pooled from three independent tests and represented as mean ± s.d., *t*-test, **** *p* < 0.0001. ns denotes not significant.

To visualize and evaluate the binding patterns of the tropomyosins on actin filaments, we used custom-made microfluidic devices (Figure 3A–C). The mixture containing either α- or β-actin monomers and labeled Tpms was incubated for 5 min, and actin polymerization was initiated by increasing the ionic strength of the mixture using NaCl salt (50 mM final concentration). The binding of the labeled Tpms to actin filaments was enhanced using the crowding effect of 10% PEG 300. The decorated filaments were non-specifically captured on the surface of the glass coverslip modified with PLL-PEG and 5% BSA and imaged using TIRF illumination (Figure 3C). We divided experiments into different conditions: binding of the same Tpm isoform labeled with either AF488 or AF568 (“control”) and binding of varying Tpm isoforms, each labeled with a different fluorophore (“competition”) (Figure 3C,D). The bound tropomyosin appeared as a diffraction-limited spot in all experimental conditions, which we call “clusters”. The images were further analyzed using the algorithm developed for this study.

Control experiments showed that the AF568-labeled Tpm and AF488-labeled Tpm were equally incorporated into clusters on the actin filament, whereas Figure 3D shows a slight increase in the occupation ratio of Tpm1.1 AF568 over the same isoform labeled with AF488 for α-actin. This finding, in addition to the biochemical assays, further validate the absence of any adverse effect of the small fluorophores AF488 and AF568 on the affinity of tropomyosins for actin. Thus, the tropomyosins with fluorophore are referred to as “labeled tropomyosin” in the “competition experiment”. Nevertheless, the “competition” experiments showed an average of 1.2, and 2.2 times more labeled Tpm1.1 than Labeled Tpm1.6 incorporated into clusters on α-actin and β-actin filament, respectively. This suggests that, at low concentrations, Tpm1.1 exhibits a higher occupation fraction for both β-actin and α-actin, indicating that it shows a higher affinity than Tpm1.6 to both actin isoforms. This is in agreement with previous studies using biochemical assays (Figure 3A–D) (15 January [19] (pp. 268–280)).

We also compared two tropomyosins using both skeletal and cytoskeletal actin filaments, and found that the distributions of inter-cluster distances were similar for all combinations of actin and tropomyosin isoforms. Notably, we observed more clusters with shorter distances between Tpm1.1s and Tpm1.6s consecutive clusters on β-actin Table 3.

### 3.4. Different Tropomyosin Isoforms Separate into Distinct Clusters

**Figure 4:**
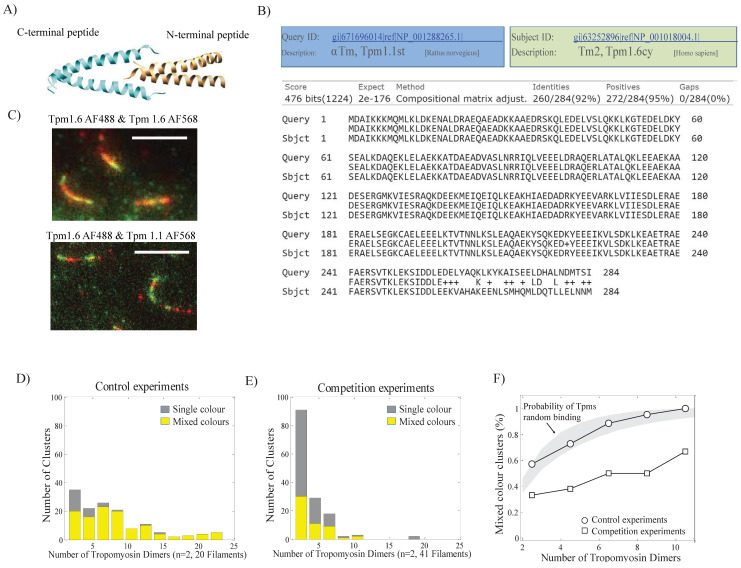
Different tropomyosin isoforms separate into distinct clusters. (**A**) An illustration of the NMR structure of the C- and N-terminal overlapping peptides with the 8–11 residue spans (adapted from Greenfield et al., 2006 [20]). (**B**) Alignment of Tpm1.1 amino-acid sequence against Tpm1.6 sequence with the 27th amino acid difference. (**C**) Representative TIRF images of similar tropomyosins mixed in the same clusters (yellow) (top panel) vs. different tropomyosin isoforms separating into distinct clusters (bottom panel). (**D**) Histograms of the number of tropomyosin dimers per cluster for single-color and mixed-color clusters for control experiments (20 filaments from two independent experiments, and competition experiments (41 filaments from two independent experiments, (**E**). The fraction of mixed-color clusters as a function of cluster size (number of tropomyosin dimers per cluster). The grey line shows the fraction of mixed clusters predicted for the random incorporation of both colors based on a binomial distribution with the experimentally determined probabilities of incorporating Tpm labeled with AF568 and Tpm labeled with AF488 (**F**).

To investigate whether the C-termini (27 C-termini amino acids) differences were sufficient to discriminate between these isoforms during the early stages of tropomyosin assembly, we quantified the composition of clusters using the method presented above. By selecting the clusters containing at least two tropomyosin dimers (i.e., at least two fluorophores), we calculated the fraction of single-color and mixed-color clusters as a function of cluster size (Figure 4F and Figure 4E, respectively). We observed that the percentage of mixed-color clusters increased with the number of tropomyosin dimers per cluster. To determine if the observed mix-color portions were due to a random probability of Tpms interacting with the actin filament, we evaluated the probability of the mixed incorporation of tropomyosins using a binomial distribution with the experimentally determined probabilities.

Our data show that, in control experiments (same isoform labeled with different colors), tropomyosin dimers bound independently to the actin filament, and did not bind as aggregates. The curve determined for the control experiment was similar to the curve predicted for the random incorporation of both colors (Figure 4D). In contrast, exposing actin filaments to different isoforms of labeled Tpm1.1 and Tpm1.6 led to a pattern of the decoration with a reduced percentage of mixed clusters compared to the control experiments (Figure 4E). This observation suggests that the two isoforms are not randomly incorporated into a growing tropomyosin strand on an actin filament. There appears to be a higher probability that the same isoform rather than different isoforms will be incorporated into the cluster during assembly (Figure 4F).

## 4. Discussion

This study prepared and labeled equimolar concentrations of rat skeletal–muscle Tpm1.1 and cytoskeletal Tpm1.6 tropomyosin molecules. Using labeled proteins, we decorated single filaments of muscle and cytoskeletal actin prior to capturing the filaments on the modified microfluidic chamber surface. Both isoforms were expressed by the same gene with only a 27 amino acid difference in their structure. An unexpected outcome of our measurements was that, despite the small differences in the structures, our results indicate that the difference was sufficient to cause the preferential neighboring binding of similar isoforms.

Tropomyosin isoforms in their native environment are sorted into specific populations of actin filaments. Some isoforms differ in only one exon, and this difference is sufficient to localize them to a particular population of actin. For example, Tm5NM1 (Tpm3.1) has been mainly observed with stress fibers and Tm5NM2 (Tpm3.2) with actin filaments associated with the Golgi apparatus [19]. Essential determinants for tropomyosin binding, polymerization, and function are the overlapping complexes between the N- and C-termini of adjacent tropomyosin dimers [5,21]. More recently, Tpm localization was also suggested to be the effect of the overlap region in an isoform-specific manner. However, differences obtained for Tpm1.1 and Tpm1.6 in intravital imaging were too small to indicate any functional differences [4]. Another study using the cross-linking of F-actin and fluorescence changes in F-actin labeled with acrylodan at Cys41 (in D-loop) did not show differences in the conformation of the C-terminal segment of F-actin in the presence of different tropomyosins with and without cofilin 1. However, they showed that tropomyosin isoforms differentially regulated cofilin-induced conformational rearrangements at longitudinal and lateral filament interfaces [22]. Thus, although the overlap complex’s importance has been well established, it has remained unclear which step in the assembly process is most affected by differences in the N- or C-terminus.

One more vital factor to consider is the binding behavior of Tpms. As long-lateral-size (approximately 30 nm) proteins, their binding behavior is described as “cooperative” [9]. Thus, a numerical approach is used to estimates the binding of Tpms to actin as an explicit function of the free Tpm concentration. The overall affinity (Kapp) of tropomyosin to the linear lattice of the actin filament is dependent on both cooperative binding behavior and affinity. Other studies suggest that the initial nucleation of tropomyosin molecules on an actin filament is affected by the cooperativity effect. These early assemblies are also indicated to cause structural changes, explaining the sorting of tropomyosin molecules in an isoform-dependent manner and the generation of homopolymers [23].

Our findings show that Tpm1.1 and Tpm1.6 can both nucleate on the same filament, while Tpm1.1 always occupies more binding sites on both α and β F-actin than Tpm1.6. We show that in the absence of a cellular regulatory environment and at the early stages of the self-assembly of tropomyosins to the actin filament, most filaments are decorated with more than one type of Tpm and form “heteropolymers”. In the physiological environment, cytoskeletal tropomyosins, in particular Tpm1.6, have been shown to appear as homopolymers [3,24,25,26]. Interestingly, in our experimental conditions, the tropomyosin concentrations (0.015–0.03 μM) used for imaging experiments were below the range at which the cooperative effect of the binding to actin filaments was observed in our co-sedimentation assays (~0.1 μM). However, in both imaging and bulk biochemistry assays, we observed a higher affinity of Tpm1.1 for actin filaments than Tpm1.6. These findings are in agreement with current models of the binding of a large ligand to extended lattice proteins. They also support the notion that the initial stage of binding of long proteins to each other is mainly affinity-dependent [27]. Together, this appears to indicate that affinity is a predominant factor in determining the binding rate of tropomyosin isoforms to actin filaments. Perhaps this is an essential early assembly step before cooperativity initiates.

A recent intravital study on salivary gland acinar cells showed that the recruitment of Tpms occurs at the same time as, or shortly after, actin assembly [21]. Similar conclusions can be drawn from the model proposed by Holmes and Lehman [28] and experimental studies by Singh and Hitchcock-DeGregori [29,30]. The direct visualization of actin filament polymerization and tropomyosin binding at the early assembly stage is essential to completing the picture. Thus, it appears that the association of tropomyosin to an actin filament can only be explained by considering the whole binding process, including (a) the initial affinity-dependent interaction of single tropomyosin dimers with the filament, (b) the subsequent head-to-tail association of tropomyosins, and (c) the copolymerization of Tpm and actin. This was captured in the current study via the combination of controlled-environment assembly using microfluidics, high spatio-temporal imaging via TIRF microscopy, and the computational detection of binding events.

## 5. Conclusions

This study explored how the tropomyosin isoforms Tpm1.1 and Tpm1.6 bind to actin filaments, finding that even small structural differences between them can lead to distinct binding preferences. While both isoforms were able to nucleate on the same filament, Tpm1.1 consistently showed a stronger affinity for actin, often occupying more binding sites than Tpm1.6. These results suggest that early binding is primarily driven by affinity rather than the cooperative effects typically seen at higher concentrations. This initial, affinity-driven interaction may be a key step in how tropomyosin and actin come together before cooperative binding takes over.

Our findings align with existing models that emphasize the importance of affinity in the early stages of tropomyosin-actin assembly and highlight the benefits of studying these interactions in a controlled environment. By using microfluidics and high-resolution imaging, we were able to capture the dynamics of this process in detail. Overall, this work sheds light on the mechanisms behind tropomyosin isoform sorting and underscores the need for further research into how these processes unfold in live cells, where homopolymer formation is more common.

## Figures and Tables

**Table 1:** Binding properties of unlabeled and labeled tropomyosin isoforms. Data are mean ± S.E. combined from 2–3 independent experiments (n = 1, Tmp1.1 Alexa Fluor 568).

Tm	K_dapp_ (µM)	α^H^
Tmp1.1	0.64 ± 0.06	3.93 ± 1.06
Tmp1.1 AF488	0.17 ± 0.02	3.52 ± 1.39
Tmp1.1 AF568	0.24 ± 0.06	3.44 ± 3.67
Tpm1.6	0.44 ± 0.07	1.86 ± 0.54
Tpm1.6 AF488	1.21 ± 0.17	2.36 ± 0.59
Tpm1.6 AF568	2.37 ± 1.38	2.03 ± 0.83

**Table 2:** Photobleaching properties of labeled tropomyosin dimers. Note that the unit for σ is pixels. Both amplitude (Amp) and integral values are in arbitrary units.

Single Molecule	Bleach Profile	Mean (σ)	Mean (Amp)	(First Step)/(Second Step)	Integral
Tpm1.1 AF568	First Step	1.56 ± 0.16	544 ± 217		10,584 ± 10,108
(n = 94)	Second Step	1.55 ± 0.14	614 ± 446	0.87	11,049 ± 4427
	Combined	1.55 ± 0.16	603 ± 395		10,171 ± 6855
Tpm1.1 AF488	First Step	1.37 ± 0.15	433 ± 218		5418 ± 6633
(n = 149)	Second Step	1.36 ± 0.13	409 ± 267	1.06	5677 ± 2209
	Combined	1.37 ± 0.16	426 ± 283		5182 ± 4061
Tpm1.6 AF568	First Step	1.55 ± 0.25	487 ± 245		9579 ± 14,245
(n = 66)	Second Step	1.60 ± 0.26	560 ± 787	0.87	13,358 ± 5036
	Combined	1.58 ± 0.23	642 ± 607		11,719 ± 9031
Tpm1.6 AF488	First Step	1.34 ± 0.13	427 ± 208		6066 ± 8451
(n = 115)	Second Step	1.35 ± 0.12	538 ± 285	0.79	6787 ± 2587
	Combined	1.35 ± 0.13	482 ± 339		6341 ± 4538

**Table 3:** Summary of quantification of labeled tropomyosins-decorated single-actin filaments. Data are pooled from three to four independent experiments and represented as median, 25% and 75% quartiles, and the total number of diffraction-limited spots.

Control Experiments				
Parameter	Tpm1.1 on β-actin	Tpm1.1 on α-actin	Tpm1.6 on β-actin	Tpm1.6 on α-actin
Ratio of Tpm in Ch1 (red, AF568)/Ch2 (green, AF488)	0.9 (0.5, 1, n = 33)	0.9 (0.6, 2, n = 44)	0.7 (0.5, 1, n = 79)	0.7 (0.5, 0.9, n = 39)
Filament length (µm)	4 (3, 6, n = 34)	5 (4, 6, n = 46)	4 (4, 6, n = 50)	7 (5, 9, n = 43)
Tpm number per cluster	3 (2, 6, n = 225)	3 (2, 6, n = 536)	2 (1, 4, n = 584)	2 (1, 4, n = 301)
Number of clusters per filament	3 (2, 4, n = 66)	5 (3, 7, n = 90)	3 (2, 5, n = 158)	3 (1, 4, n = 84)
Cluster distances (µm)	0.6 (0.5, 1, n = 157)	0.5 (0.4, 0.7, n = 442)	0.6 (0.4, 1, n = 413)	0.9 (0.5, 2, n = 202)
**Competition Experiments**				
**Parameter**	**α-actin**		**β-actin**	
Ratio of Tpm 1.1 vs. Tpm 1.6	1.2 (0.9, 2, n = 57)		2.2 (1, 3, n = 38)	
Filament length (µm)	5 (4, 6, n = 64)		5 (4, 6, n = 43)	
Tpm number per cluster (Tpm1.1)	2 (1, 4, n = 229)		5 (2, 13, n = 219)	
Tpm number per cluster (Tpm1.6)	2 (1, 3, n = 176)		2 (1, 5, n = 229)	
Number of clusters per filament (Tpm1.1)	4 (2, 5, n = 62)		5 (3, 9, n = 41)	
Number of clusters per filament (Tpm1.6)	2 (1, 4, n = 62)		5 (4, 8, n = 41)	
Cluster distances (µm)	0.8 (0.5, 1, n = 146)		0.49 (0.4, 0.7, n = 179)	

## Data Availability

The code used to collect and analyze the data discussed in this paper is available upon request from the corresponding author.

## Supplementary Materials

The following supporting information can be downloaded at: https://www.mdpi.com/article/10.3390/biom14101240/s1. Figure S1: Performance of the algorithm for detection and quantification of the number of molecules in punctate signals along filaments. (A) Iterative detection and depletion of the signal with the highest amplitude in a region of interest. Line profile and a corresponding image containing nine simulated diffraction-limited spots (top) with an amplitude of 344 ± 43 a.u. and a width (σ) of 1.69 ± 0.001 pixels. The first iteration through the algorithm leads to detecting the highest peak and its replacement with average background noise (bottom). (B) Accuracy of the technique for determining the number of molecules from simulated diffraction-limited spots containing 1–20 fluorophores. (C) 2D side-view intensity profiles and corresponding TIRF images (from the experiment) of surface-bound Tpm molecules (top) and Tpm molecules are decorating a surface-bound actin filament (bottom) with a signal-to-noise ratio (SNR) of ~17. (D) Image of simulated diffraction-limited spots with different SNRs (left). The violin plots show the distribution of the number of recovered single particles from a simulated image with 30 spots with a given SNR (inside axes numbers represent the number of peaks). (E) Representative simulated images are depicting sets of two particles with a defined separation (1–3.5 pixel(s) × σ (σ = 1.69 pixels). (F) Violin plots showing the distribution of the diffraction-limited spot detection accuracy with different distances from each other (inside axes numbers represent the number of pairs of peaks). Figure S2: (A) The simulated image contains 50 consecutive diffraction-limited spots (amplitude 345.47 ± 0.42 a.u. and sigma 1.6902 ± 0.0010 pixels).

## References

[1] Obeidy P., Sobey T.L., Pandzic E., Nicovich P.R., Coster A., Gunning P., Böcking T. (2015). Actin Tropomyosin Assembly Intermediates. Biophys. J..

[2] Gunning P., O’Neill G., Hardeman E. (2008). Tropomyosin-based regulation of the actin cytoskeleton in time and space. Physiol. Rev..

[3] Gimona M., Watakabe A., Helfman D.M. (1995). Specificity of dimer formation in tropomyosins: Influence of alternatively spliced exons on homodimer and heterodimer assembly. Proc. Natl. Acad. Sci. USA.

[4] Åšliwiå„Ska M., Moraczewska J. (2013). Structural differences between C-terminal regions of tropomyosin isoforms. PeerJ.

[5] Moraczewska J., Nicholson-Flynn K., Hitchcock-DeGregori S.E. (1999). The Ends of Tropomyosin Are Major Determinants of Actin Affinity and Myosin Subfragment 1-Induced Binding to F-Actin in the Open State. Biochemistry.

[6] Mak A.S., Smillie L.B. (1981). Non-polymerizable tropomyosin: Preparation, some properties and F-actin binding. Biochem. Biophys. Res. Commun..

[7] Hammell R.L., Hitchcock-DeGregori S.E. (1996). Mapping the functional domains within the carboxyl terminus of al-pha-tropomyosin encoded by the alternatively spliced ninth exon. J. Biol. Chem..

[8] von der Ecken J., Müller M., Lehman W., Manstein D.J., Penczek P.A., Raunser S. (2014). Structure of the F-actin–tropomyosin complex. Nature.

[9] Tobacman L.S. (2008). Cooperative binding of tropomyosin to actin. Adv. Exp. Med. Biol..

[10] Axelrod D. (2001). Total Internal Reflection Fluorescence Microscopy in Cell Biology. Traffic.

[11] Zhang H., Guo P. (2014). Single molecule photobleaching (SMPB) technology for counting of RNA, DNA, protein and other molecules in nanoparticles and biological complexes by TIRF instrumentation. Methods.

[12] Mutch S.A., Fujimoto B.S., Kuyper C.L., Kuo J.S., Bajjalieh S.M., Chiu D.T. (2007). Deconvolving Single-Molecule Intensity Distributions for Quantitative Microscopy Measurements. Biophys. J..

[13] Peterson E.M., Harris J.M. (2009). Quantitative Detection of Single Molecules in Fluorescence Microscopy Images. Anal. Chem..

[14] Monteiro P., Lataro R., Ferro J., Reinach F.d.C. (1994). Functional alpha-tropomyosin produced in Escherichia coli. A dipeptide extension can substitute the amino-terminal acetyl group. J. Biol. Chem..

[15] Janco M., Bonello T.T., Byun A., Coster A.C.F., Lebhar H., Dedova I., Gunning P.W., Böcking T. (2016). The impact of tropomyosins on actin filament assembly is isoform specific. BioArchitecture.

[16] Kremneva E., Boussouf S., Nikolaeva O., Maytum R., Geeves M.A., Levitsky D.I. (2004). Effects of two familial hypertrophic cardiomyopathy mutations in alpha-tropomyosin, Asp175Asn and Glu180Gly, on the thermal unfolding of actin-bound tropomyosin. Biophysical. J..

[17] Betcher-Lange S.L., Lehrer S.S. (1978). Pyrene excimer fluorescence in rabbit skeletal alphaalphatropomyosin labeled with N-(1-pyrene)maleimide. A probe of sulfhydryl proximity and local chain separation. J. Biol. Chem..

[18] Orzechowski M., Moore J.R., Fischer S., Lehman W. (2014). Tropomyosin movement on F-actin during muscle activation explained by energy landscapes. Arch. Biochem. Biophys..

[19] Percival J.M., Hughes J.A.I., Brown D.L., Schevzov G., Heimann K., Vrhovski B., Bryce N., Stow J.L., Gunning P.W. (2004). Targeting of a Tropomyosin Isoform to Short Microfilaments Associated with the Golgi Complex. Mol. Biol. Cell.

[20] Greenfield N.J., Huang Y.J., Swapna G., Bhattacharya A., Rapp B., Singh A., Montelione G.T., Hitchcock-DeGregori S.E. (2006). Solution NMR structure of the junction between tropomyosin molecules: Implications for actin binding and regulation. J. Mol. Biol..

[21] Masedunskas A., Appaduray M.A., Lucas C.A., Cagigas M.L., Heydecker M., Holliday M., Meiring J.C.M., Hook J., Kee A., White M. (2018). Parallel assembly of actin and tropomyosin, but not myosin II, during de novo actin filament formation in live mice. J. Cell Sci..

[22] Ostrowska-Podhorodecka Z., Śliwinska M., Reisler E., Moraczewska J. (2020). Tropomyosin isoforms regulate cofilin 1 activity by modulating actin filament conformation. Arch. Biochem. Biophys..

[23] Śliwińska M., Skórzewski R., Moraczewska J. (2008). Role of Actin C-Terminus in Regulation of Striated Muscle Thin Filament. Biophys. J..

[24] Coulton A.T., East D.A., Galinska-Rakoczy A., Lehman W., Mulvihill D.P. (2010). The recruitment of acetylated and unacetylated tropomyosin to distinct actin polymers permits the discrete regulation of specific myosins in fission yeast. J. Cell Sci..

[25] Tojkander S., Gateva G., Schevzov G., Hotulainen P., Naumanen P., Martin C., Gunning P.W., Lappalainen P. (2011). A Molecular Pathway for Myosin II Recruitment to Stress Fibers. Curr. Biol..

[26] Stehn J.R., Haass N.K., Bonello T., Desouza M., Kottyan G., Treutlein H., Zeng J., Nascimento P.R., Sequeira V.B., Butler T.L. (2013). A Novel Class of Anticancer Compounds Targets the Actin Cytoskeleton in Tumor Cells. Cancer Res..

[27] McGhee J.D., von Hippel P.H. (1974). Theoretical aspects of DNA-protein interactions: Co-operative and non-co-operative binding of large ligands to a one-dimensional homogeneous lattice. J. Mol. Biol..

[28] Holmes K.C., Lehman W. (2008). Gestalt-binding of tropomyosin to actin filaments. J. Muscle Res. Cell Motil..

[29] Smith D., Geeves M. (2003). Cooperative Regulation of Myosin-Actin Interactions by a Continuous Flexible Chain II: Actin-Tropomyosin-Troponin and Regulation by Calcium. Biophys. J..

[30] Singh A., Hitchcock-DeGregori S.E. (2006). Dual Requirement for Flexibility and Specificity for Binding of the Coiled-Coil Tropomyosin to Its Target, Actin. Structure.

