# Supplementary material for: Tropomyosin Isoforms Segregate into Distinct Clusters on Single Actin Filaments
Source: Biomolecules. 2024 Sep 30;14(10):1240. doi: 10.3390/biom14101240 (PMC11506546; doi:10.3390/biom14101240)
Supplement: Supplementary file 1 [file biomolecules-14-01240-s001.zip › biomolecules-3012075-supplementary materials.pdf]

# Supplementary materials

## Title

Tropomyosin isoforms segregate into distinct clusters on single actin filaments

## Authors

Peyman Obeidy[1,2]*, Thomas Sobey[1], Philip R. Nicovich[3], Adelle C. F. Coster[4], Elvis Pandzic[5]*

## Affiliation

[1] Single Molecule Science, School of Medical Sciences, UNSW, Sydney, NSW, 2052, Australia.
[2] School of Biomedical Engineering, Faculty of Engineering, The University of Sydney, Affiliated with Sydney Nano Institute, Sydney Manufacturing Hub Camperdown, NSW, 2006, Australia.
[3] Allen Institute for Brain Science, Seattle, WA, USA
[4] School of Mathematics & Statistics, Faculty of Science, UNSW Sydney, NSW, 2052, Australia.
[5] Katharina Gaus Light Microscopy Facility, Mark Wainwright Analytical Centre, UNSW Sydney, Sydney, NSW, 2052, Australia.

*Corresponding author. Email: peyman.obeidy@sydney.edu.au, e.pandzic@unsw.edu.au

# Introduction

The custom-written single-molecule algorithm developed for quantifying the number of single-molecule of tropomyosins on actin filaments in this study was verified using simulated data. For example, diffraction-limited spots were simulated and recovered from images with varying background noise or located at different distances from one another. These findings showed that our custom-written algorithm has 80% accuracy in estimating the number of single particles in the experimental data.

# Results

The algorithm examines each diffraction-limited spot on a given filament in one iteration. In each iteration, the highest peak is located, analyzed and subtracted from the image. The coding procedure was deciphered when each iteration was outputted into an image. The outcome showed that the diffraction-limited spot with the highest amplitude was initially selected as the first point for analysis, and this process continued until reaching the background signal (Fig. S1A). Further analysis using a simulated image with multiple diffraction-limited spots accumulated in each position demonstrated that the code could recover 1-7 diffraction-limited spots with no error and 8-15 particles with above 88% accuracy (Fig. S1B).

Next, we calculated the signal to noise ratio (SNR) in experimental data as a reference point. We selected random experimental data for the reference image containing images of labeled actin filaments decorated with tropomyosins. The SNR of the first frame in the experimental data for filament images and the single tropomyosin regions were 19.8±3.7 (n=5) and 17±2 (n=7 regions), respectively (Fig. S1C). Subsequently, we investigated the algorithm's ability to recover diffraction-limited spots in simulated images with varying SNR. Our findings demonstrated 84.4±4% and 90±4% accuracy in recovering a single particle embedded in SNR of 13.3 17.8, respectively (Fig. S1D, left panel). The recovery accuracy in perfect data (i.e. SNR of 55.3) was 93±2% (Fig. S1D, right panel).

Analyzing an elongated diffraction-limited spot requires cutting an ROI of 4x4 pixels around each spot. These occasionally leaves small shoulders behind. The algorithm is equipped with a merging function to marry particles where the centres of the diffraction-limited spots are closer than half of the sigma (sigma is ~1.69 pixels). Furthermore, the precision of the merging function was examined by evaluating the recovery of the spots with varying distances from one another. These data were recovered with 90% accuracy when two diffraction-limited spots were located 3.3 × δ (δ= Gaussian e^-2 radius is set on 1.69 pixels) apart. This value reached 98% accuracy for two diffraction-limited spots with distances equal to and greater than 3.5 × δ (Fig. S1E-F).

Images of diffraction-limited spots representing single particles with known parameters were generated and analyzed to validate the performance of our custom-written algorithm. To determine whether the code can analyze long filaments, we sought to evaluate the recovery of 50 consecutive diffraction-limited spots on one filament. The length of this stimulated filament (472 pixels, ~50 µm) is almost ten times longer than the filaments observed in experimental data (~5 µm). Usually, a filament in experimental data is coated with no more than 25 diffraction-limited spots (referred to as clusters of tropomyosins molecules in the experimental data). Analyzing this long filament, we demonstrated that the algorithm is well equipped to detect information from a long filament (Fig. S2A).

These findings showed that the algorithm is equipped with a merging function to marry close particles belonging to one signal from an elongated diffraction-limited spot.

# Methods

To generate simulated images containing diffraction-limited spots, the "spotmaker" function in MATLAB® was used (written by Tristan Ursell (2012). The images were analyzed with the custom-written single-molecule algorithm.

**Detection of single particles on a long filament**

An image containing 50 consecutive diffraction-limited spots was generated. Each diffraction-limited spot has an airy profile with amplitude and sigma of 345.47±0.42 and 1.6902±0.0010, respectively.

**Visualizing the algorithm process**

To illustrate the process of the algorithm through each iteration, we simulated an image of nine consecutive diffraction-limited spots with different amplitudes (344±43) and constant sigma (1.69±0.001) values. The algorithm is set to operate by finding the local maximum intensity in the fluorescence image and subsequently fit it with a 2D Gaussian function in its neighbourhood of 4 by 4 pixels. The fitted Gaussian is then subtracted from the image. The process is repeated for the second iteration until the signal level reaches background noise (i.e. the standard deviation of the signal is equal to or smaller than the background noise).

**Detection of the number of single particles per spot in simulated images**

The algorithm accuracy in recovering the number of simulated diffracting-limited spots per spot location was evaluated by analyzing images containing nine simulated diffracting-limited spots. Each simulated spot was designed to have an airy pattern with the amplitude and the sigma of 345.4±5, 1.69±0.2, respectively. The background noise of each image was set to 10±5 (a.u.). These values were estimated based on the preliminary observation in the experimental images. Approximately 2-10 diffracting-limited particles (Tpms) were detected in each cluster of proteins on a given filament (data not shown).

**Detection of the single particles in the simulated image with different signal to noise ratio**

Recovering simulated diffraction-limited spots embedded in different signal to noise ratios was also tested by simulating 30 consecutive diffraction-limited spots in images with different SNR (image size 40×325 pixels matrix). SNR was set in an image by modulating the "noise-amplitude" parameter in the "Spotmaker" function while keeping the amplitude of diffraction-limited spot constant at 345.45±0 (a.u.). Subsequently, the SNR value in each simulated image was determined by the following equation (1):

SNR= (mean (signal mask)-mean (background mask))/STD (background mask) (S1)

where a rectangular ROI defines the "signal mask" in the middle of the image containing the diffraction-limited spots and "background mask" is the ROI from the whole image excluding the signal mask. For the signal mask, the mean of the 30 peaks was determined by their local-maxima amplitude.

**Merging of the single particles located within a certain distance from each other**

To evaluate the merging function in our algorithm, we varied the distance between diffraction-limited spots and set them (Gaussian e^-2 radius) between 1.5 and 3.5 pixels apart. Every single particle has amplitude, sigma and image noise of 345.4±5, 1.69±0.2 and 10±5 (a.u.), respectively. The merging function marries particles where the centres of the diffraction-limited spots are closer than half of the sigma (sigma is ~1.69 pixels).

**Supplementary data**

**Figure S1.** Performance of the algorithm for detection and quantification of the number of molecules in punctate signals along filaments. (A) Iterative detection and depletion of the signal with the highest amplitude in a region of interest. Line profile and a corresponding image containing nine simulated diffraction-limited spots (top) with an amplitude of 344±43 a.u. and a width (σ) of 1.69±0.001 pixels. The first iteration through the algorithm leads to detecting the highest peak and its replacement with average background noise (bottom). (B) Accuracy of the technique for determining the number of molecules from simulated diffraction-limited spots containing 1-20 fluorophores. (C) 2D side-view intensity profiles and corresponding TIRF images (from the experiment) of surface-bound Tpm molecules (top) and Tpm molecules are decorating a surface-bound actin filament (bottom) with a signal-to-noise ratio (SNR) of ~17. (D) Image of simulated diffraction-limited spots with different SNRs (left). The violin plots show the distribution of the number of recovered single particles from a simulated image with 30 spots with a given SNR (inside axes numbers represent the number of peaks). (E) Representative simulated images are depicting sets of two particles with a defined separation (1-3.5 pixel(s)×σ (σ=1.69 pixels). (F) Violin plots showing the distribution of the diffraction-limited spot detection accuracy with different distances from each other (inside axes numbers represent the number of pairs of peaks).

**Figure S2.** (A) The simulated image contains 50 consecutive diffraction-limited spots (amplitude 345.47±0.42 a.u. and sigma 1.6902±0.0010 pixels). (B) Selection of the diffraction-limited spot with the highest amplitude value displaying the algorithm procedure. (C) Scatter plot with a bar graph of raw data retrieved from 1-20 diffraction-limited spot(s) in each location.
