# Supplementary figures and images for: Tropomyosin Isoforms Segregate into Distinct Clusters on Single Actin Filaments
Source: Biomolecules. 2024 Sep 30;14(10):1240. doi: 10.3390/biom14101240 (PMC11506546; doi:10.3390/biom14101240)

**Figure S1**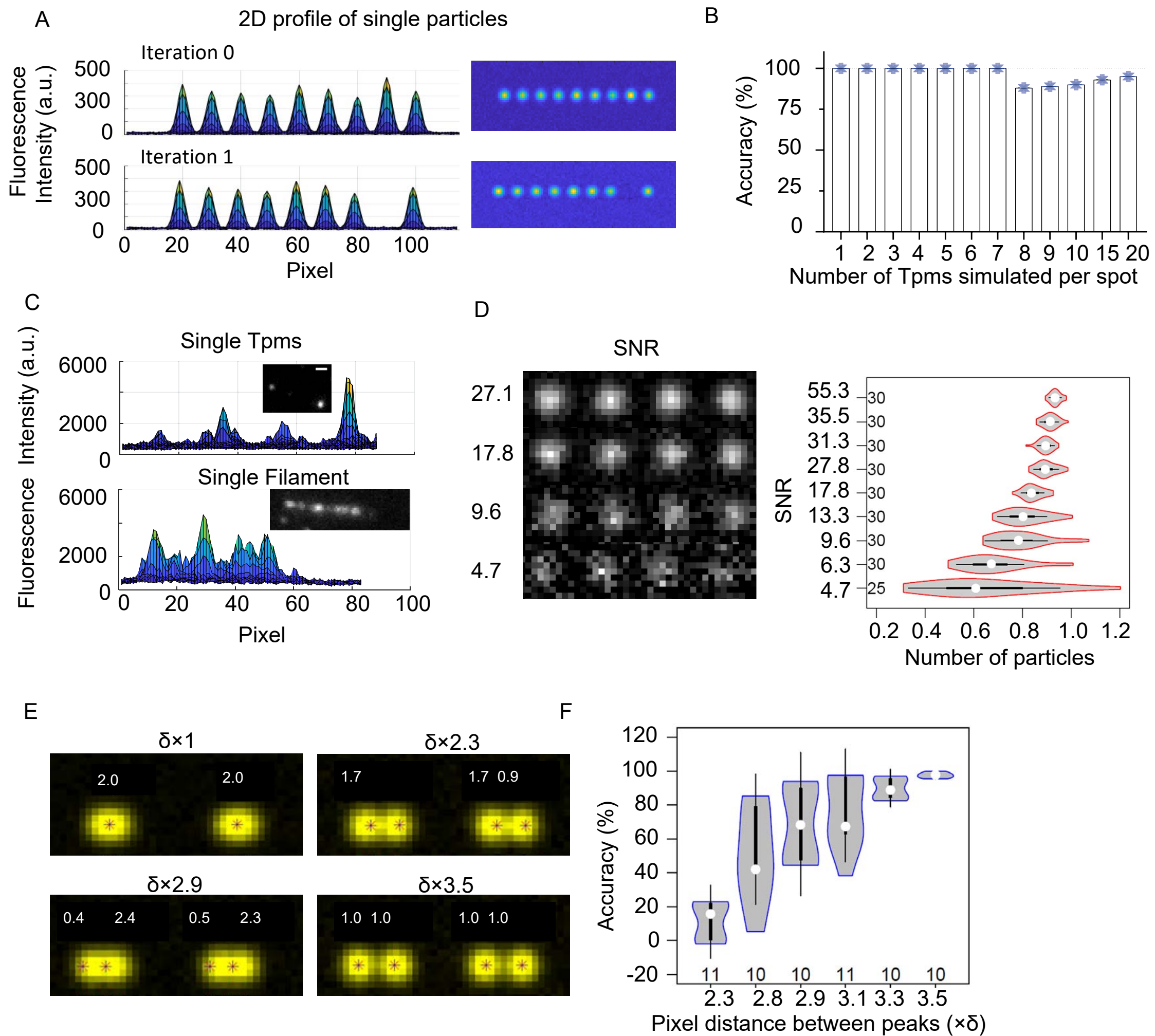

Figure S2

A

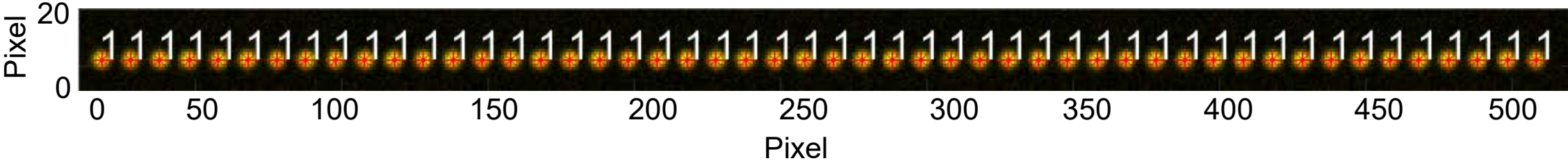

Supplement: Supplementary file 1 [file biomolecules-14-01240-s001.zip › biomolecules-3012075-supplementary figures.pdf]
